# Effect of characteristics of different wheat flours on the quality of fermented hollow noodles

**DOI:** 10.1002/fsn3.2442

**Published:** 2021-07-19

**Authors:** Xiaoqing Xiong, Chong Liu, Mengkun Song, Xueling Zheng

**Affiliations:** ^1^ College of Food Science and Engineering Henan University of Technology Zhengzhou P. R. China

**Keywords:** dough quality, fermentation, noodle quality, relationship, wheat flour characteristics

## Abstract

Hollow noodles, also known as Kongxin noodles in China, are traditionally hand‐made noodles produced by spontaneous fermentation. It is easy to cook, nutrient‐rich, and delicious. However, it is difficult to realize industrial production by spontaneous fermentation due to its complexity. More recently, new techniques have emerged for producing such noodles industrially using commercial yeasts. However, there are no reports on how to choose the raw materials for making fermented hollow noodles. Therefore, the suitability of eleven local varieties of wheat flour was determined by evaluating their physicochemical, rheological properties, and pasting properties. Flour and dough properties of wheat flour were also correlated with the quality characteristics of hollow noodles. The correlation coefficient data indicated that the color score was negatively correlated with ash content and positively correlated with starch content. Different from ordinary dried noodles, a negative correlation was observed between cooking time (CT) and protein content. Water absorption (NWA) of hollow noodles was negatively affected by extensograph properties. Water absorption of flour (FWA) and extensibility (E) were found to be highly correlated to hollow rate (Hol‐R), indicating that these two indexes could predict the fermentation status of hollow noodles. Results showed that wheat flours with higher swelling index of glutenin (SIG), FWA, E, and pasting temperature (PT) had better dough fermentation power and stability and thus were beneficial to the production of high‐quality hollow noodles. This study provides a simple method for the industrial production of hollow noodles and provides a basis for the selection of raw materials for their production.

## Graphical abstract

Water absorption of flour (FWA) and extensibility (E) were found to be highly correlated to hollow rate (Hol‐R), indicating that these two indexes could predict the fermentation status of hollow noodles. Results showed that hollow noodles with stable fermentation and high quality demanded wheat flours with high swelling index of glutenin (SIG), FWA, E, and pasting temperature (PT).

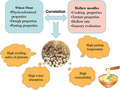

## 1 INTRODUCTION

Noodles, as the low‐cost, convenience staple foods, are prepared mostly from wheat flour. Nowadays, consumers pay more attention to nutritional and healthy noodle products and require the noodles with ready‐to‐eat convenience and superior flavor and taste. Fermentation can convert wheat flour dough into inviting, toothsome and digestible products, and this technology is mainly used in the production of bread and steamed bread. However, in order to improve the nutrition, functional, sensory, and technological characteristics of noodle products, fermentation step as an innovative strategy was applied to pasta making (Montemurro et al., 2019).

Recently, studies were mainly focused on using non‐conventional flours (e.g., legumes and pseudo‐cereals) to improve the peculiar flavor, texture properties, and nutritional value of fermented noodles or pasta (Li et  al., 2018; Lorusso et al., 2017; Marengo et al., 2015). Additionally, sourdough fermentation is one of the oldest biotechnologies widely employed in bread and steamed bread production, as it decreases glycemic index, phytate content, and trypsin inhibitors (Coda et al., 2010, 2011; Moroni et al., 2012). To enhance nutritional and functional properties of pasta, researchers argued that sourdough‐fermented ingredients are potentially applicable in pasta making (Montemurro et al., 2019). Specifically, pasta prepared by liquid sourdough decreased the slowly digestible starch content while increased the contents of inaccessible digestible starch and retrograded starch in noodles (Fois et al., 2017). In general, fermentation as an efficient and innovative tool can meet the requirements of modern consumers for nutritionally balanced and functional foods. As a fermented noodle product, hollow noodles have been popular in China for hundreds of years.

Hollow noodles with smooth surfaces and small holes like silver wire inside are traditional and unique Chinese noodles. The hollow noodles are convenient, easy to cook, nutritious, and delicious, thus deeply loved by children and the elderly. At present, hollow noodles are mainly hand‐made and spontaneously fermented based on previous experience by a complex microbiome dominated by yeast population. After repeated fermentation and stretching (necessary steps for making hollow noodles), a large number of microholes appear, which confers the hollow noodles with pleasant fermented flavor and good rehydration. Nevertheless, the manufacturing procedures of hand‐made hollow noodles are various, complicated (up to 20 steps), and time‐consuming (up to 70 hr). The fermentation process is also vulnerable to the weather. Therefore, the production of hand‐made hollow noodles is limited and it is difficult to carry out large‐scale industrialization.

More recently, new techniques have emerged for producing hollow noodles industrially using commercial yeasts. Yihai Kelly Group (China) has launched an industrial fermentation type hollow noodles which have been applied for a patent (Qiu et al., 2019). When the dough is mixing, yeast is added. After cutting, noodles are fermented in the pre‐drying process. The key technology includes two fermentation stages (the first stage: humidity is 85%–95%, temperature is 20–35℃, and fermentation time is 0.5–1.5 hr; the second stage: humidity is 80%–85%, temperature is 33–40℃, and fermentation time is 1.0–2.0 hr). However, to date, the research on the hollow noodles has not been reported in detail. Therefore, raw materials, formulation, technology, and its quality formation mechanism need to be further investigated.

Wheat flour plays a dominant role in the quality of noodles. The physicochemical (protein, ash, and damaged starch content) and farinographic properties (water absorption, dough stability) of wheat flour were highly related with white salted noodle quality (Lindsay, 2012; Noda et al., 2001; Park & Baik, 2002; Yun et al., 1996). Much research had been established to study the effects of flour and dough properties on the quality of noodles. For example, firmness of the noodles was positively correlated with wet gluten, dry gluten, and protein content (*r* = .676, .640, and .682, *p* < .05, respectively) (Kaur et al., 2015). Presence of higher protein content might also result in lower swelling of starch granules that prevented the excessive leaching of amylose during cooking (Sissons & Batey, 2003). High peak viscosity and breakdown had positive effects on the appearance, texture (stickiness and elasticity), smoothness, and taste of dry white Chinese noodles (Liu et al., 2003). However, the production process of fermented noodles is unique, which is mainly reflected in the fact that yeast is required to produce gas and porous structure in noodles. In order to retain gas in the noodles, wheat flour with higher strength of gluten may be needed. On the other hand, different from the well‐developed bread dough with high moisture content (40%–55%), an underdeveloped crumbly dough and dough sheet with low moisture content (28%–38%) were, respectively, formed in the mixing and sheeting processes of noodle making. And yeast growth, gas production, and gas‐holding capacity in noodle dough may be different from that of bread dough. Therefore, the quality index of wheat flour for fermented noodles is expected to be different from that of other fermented products such as bread and steam bread. However, so far, there is a lack of research on the quality requirements of special raw materials for the new type of industrial fermented noodles–hollow noodles. It is necessary to find out which quality indexes of wheat flour can predict the quality of hollow noodles well.

In this work, the characteristics of 11 brands of wheat flour used for hollow noodles making were evaluated by physicochemical, rheological, and paste properties; then, a simple and industrializable production process is adopted for hollow noodles making and the correlation between characteristics of wheat flour and quality of hollow noodles were investigated. The objective was to provide a theoretical basis for the establishment of the raw material quality standard for making hollow noodles.

## 2 MATERIALS AND METHODS

### 2.1 Materials

Eleven different kinds of commercial wheat flours were obtained from the market including Xiangxue high gluten wheat flour (Xiangxue HG) (Shenyang Xiangxue Flour Co. Ltd), Xiangmanyuan high‐quality wheat flour (Xiangmanyuan) (Yihai Kerry Co. Ltd), Xuejian multi‐purpose wheat flour (Xuejian MP) (Henan Xuejian Industrial Co. Ltd), Sifeng fine wheat flour (Sifeng) (Xinxiang Sifeng Powder Industry Co. Ltd), Xuejian wheat core flour (Xuejian WC) (Henan Xuejian Industrial Co. Ltd), Sany original wheat flour (Sany) (Zhengzhou Tiandiren Flour Industry Co. Ltd), Golden Arowana multi‐purpose wheat flour (Golden Arowana) (Yihai Kerry Co. Ltd), Wudeli high gluten wheat flour (Wudeli) (Wudeli Group Xinxiang Flour Co. Ltd), Jinyuan refined wheat flour (Jinyuan) (Henan Jinyuan Grain and Oil Co. Ltd), Fengzheng low gluten wheat flour (Fengzheng LG) (Weifang Fengzheng Flour Co. Ltd), Fengzheng high gluten wheat flour (Fengzheng HG) (Weifang Fengzheng Flour Co. Ltd). Active dry yeast (Angel Yeast Co. Ltd) was purchased from a local supermarket. All other reagents used in this experiment were of analytical grade.

### 2.2 Physicochemical properties of wheat flour

The analyses were performed according to the method of AACC (2000). The flours were analyzed for moisture (AACC 44–19), protein (AACC 46–15), ash (AACC 08–01), wet gluten and its index (AACC 38–12), and Zeleny sedimentation value (AACC 56–61). The swelling index of glutenin in SDS solvent was carried out according to the previous study (Wang & Kovacs, 2002, 2002a). The color of different types of wheat flours, determined by L^*^, a^*^, and b^*^ values, was performed in terms of the method of Kaur et al. (2015). Each measurement was repeated at least three times.

### 2.3 Dough rheological properties of wheat flour

Farinograph properties of flour were analyzed by the AACC method 54–22 (2000) using a 300 g Brabender Farinograph equipment (Brabender GmbH & Co KG). Extensograph properties of flour were analyzed by the AACC method 54–10 (2000) using Brabender Farinograph and Extensograph (Brabender GmbH & Co KG). The data were provided with the energy value (area under the curve, cm^2^), the resistance to extension (R, BU), the dough extensibility (E, mm), and R/E value at 45 min.

### 2.4 Pasting properties of wheat flour

Pasting properties of different wheat flours were evaluated by a Rapid Visco‐Analyzer (RVA‐4, Newport Scientific Instruments). Flour (3 g, 14% moisture basis) and 25 ml distilled water were added to an aluminum RVA sample canister and then run a heating and cooling cycle program: a heating step, from 50 to 95℃ at 12℃/min (after an equilibration time of 1 min at 50℃), a holding phase at 95℃ for 5 min, a cooling step from 95 to 50℃ at 12℃/min, and a holding phase at 50℃ for 2 min.

### 2.5 Preparation of hollow noodles

The preparation process of hollow noodles in this study was not as complicated as traditional method and was more suitable for industrial production. The ingredients used in the preparation of the hollow noodles were 200 g wheat flour, 60 g distilled water, and 2 g yeasts. First, the yeasts and distilled water were mixed in a beaker and then preserved heat at 38℃ in a water bath for 10 min. Dough was formed using a mixer (JHMZ 200, East Fude Technology Development Center, Beijing) which was operated at low speed for 3 min and medium speed for next 4 min. After mixing, the prepared dough was placed in a plastic bag and rest for 15 min in a thermostated container at 25℃. The noodle dough was then passed through a laboratory JMTD 168/140 noodle machine (East Fude Technology Development Center, Beijing, China) at the gap of 3 mm for 4 passes. Each pass included folding the dough sheet to double the thickness. After being doubled folded, the roll gap was adjusted to 2.0 mm for another pass. Following that, five more passes were made to reduce the gap progressively to 1 mm. Finally, the obtained sheet was cut into rectangles of dimensions of 25 cm ×0.2 cm ×0.1 cm. The resultant noodle strands were hung on rods and placed in an incubator at 38℃ and 85% RH for 2 hr (a fermentation step), then reduced relative humidity, and dried in incubator in two stages. In the first stage, the temperature was kept at 38℃ and relative humidity was at 65% for 10 hr. In the second stage, the temperature was kept at 25℃ for 4 hr and the relative humidity was at 65%. Finally, the moisture content of dried hollow noodles was at 12%–13%. The dried hollow noodles were cut into the length of 10 cm and stored in sealed plastic bags for the next studies.

Hollow noodles were prepared on two different days (2 independent trials), and two bags of hollow noodles were prepared for each flour type.

### 2.6 Cooking and texture properties of hollow noodles

Cooking properties were determined by the method of Liu et al. (2018). Texture profile analysis (TPA) and tensile test (within 5 min after cooking) were determined by using a TA‐XT 2i texture analyzer (Scarsdale, NY, USA; Stable Micro Systems, Surrey, UK) with the HDP/PFS and A/SPR probe according to the method of Lu et  al. (2009). Each sample was measured for five times. The maximum and minimum values were removed, and the result was the average of the remaining values.

### 2.7 Hollow rate of hollow noodles

The fermentation state of hollow noodles was determined by hollow rate, and not all noodles were fermented into hollow ones. Satisfactory fermentation states were generally along with high hollow rates. Fifty noodles were randomly selected from each type of wheat flour and the number of hollow noodles (L) was counted. The hollow rate of hollow noodles was expressed as: % hollow rate of hollow noodles =L/50 × 100.

### 2.8 Sensory evaluation of hollow noodles

Ten trained food engineering students participated in the sensory evaluation of the hollow noodles, including color, firmness, viscoelasticity, smoothness, and flavor. A 9‐point scale (1 = dislike extremely and 9 = like extremely) (ISO 11136:2014) was used for this evaluation. During sensory evaluation, each sample was placed in the same plate coded with a digit number, and drinking water was provided to clean mouths during tasting.

### 2.9 Statistical analysis

The data reported in all tables are averages of at least three replications. Pearson correlation (*r*) between different properties and analysis of variance (ANOVA) between different wheat flours by Duncan's test was conducted using software 19.0 (SPSS Inc.). Significance was defined at *p* < .05.

## 3 RESULTS AND DISCUSSION

### 3.1 Physicochemical properties

Wheat flours showed a variation in the quality characteristics (Table 1). The moisture content was observed to be 12.36%–14.79%, and the ash content was 0.35%–0.60%. Ash content is related to the degree of flour refinement, but must be cautious to use it as a flour refinement indicator, because the ash content is different among wheat varieties (Symons & Dexter, 1991). Wheat starch, occupied about 70% of wheat flour, is contributed to the quality of noodle products (Guo et al., 2003). Much research had also shown that the components of flour (protein, starch, fat, ash, etc.) are inextricably linked with the quality of noodles, especially the protein and starch have the greatest impact on the quality of noodles (Lindsay, 2012; Liu et al., 2003). Starch content ranged from 76.90% to 83.03%, and there was no significant difference among the samples (*p* > .05). The protein content, wet gluten content (WG), and gluten index (GI) were in the range from 8.35% to 12.48%, 21.89% to 33.13%, and 52.38% to 95.26%, respectively. GI indicates the information about quantity and quality of wet gluten, which is strongly influenced by environmental factors (ŠIMIĆ et al., 2006). The Sany flour (12.48%) displayed the highest protein content while Fengzheng HG (8.35%) showed the lowest, but Fengzheng HG had the highest GI (95.26). This result indicated that GI did not depend on protein content. Zeleny sedimentation value (ZeSV) varied from 51.75 ml in Xuejian WC to 38.25 ml in Fengzheng HG. The values of SIG ranged from 367.04 to 452.57. SIG can be used to predict insoluble glutenin content and wheat gluten strength (Wang & Kovacs, 2002). L^*^, a^*^, and b^*^ values of different flours ranged from 98.64 to 100.02, −0.02 to 0.34, and 6.44 to 8.64, respectively. Wudeli flour had higher L^*^ value and lower a^*^ value as compared to other flours, indicating the highest brightness and the lowest redness were observed for Wudeli flour. Sifeng flour showed the highest b^*^ value, indicating more yellowness was observed for this flour. The presence of high levels of carotenoids and xanthophylls might be responsible for the increased yellowness (Kaur et al., 2015).

**TABLE 1:** Physicochemical properties of different wheat flours

Varieties	Moisture (%)	Ash (%)	Starch (%)	Protein (%)	WG (%)	GI	ZeSV (mL)	SIG	L^*^	a^*^	b^*^
Xiangxue HG	13.85 ± 0.01^d^	0.40 ± 0.01^de^	80.74 ± 1.90^a^	11.38 ± 0.13^b^	31.72 ± 0.62^abc^	52.38 ± 0.68^f^	41.25 ± 0.25^d^	381.15 ± 1.36^de^	99.60 ± 0.00^c^	0.10 ± 0.06 cd	7.34 ± 0.12^e^
Xiangmanyuan	14.79 ± 0.00^a^	0.43 ± 0.02^cde^	81.99 ± 0.15^a^	11.07 ± 0.01^b^	30.97 ± 0.50 cd	61.01 ± 2.60^e^	42.25 ± 0.75^d^	393.47 ± 9.20^d^	99.34 ± 0.08^e^	0.16 ± 0.05^c^	7.78 ± 0.10^bc^
Xuejian MP	13.25 ± 0.02^e^	0.58 ± 0.01^a^	76.92 ± 0.81^a^	12.13 ± 0.09^a^	31.63 ± 0.23^bc^	86.90 ± 1.32^bc^	50.75 ± 0.75^a^	433.98 ± 1.40^abc^	99.32 ± 0.04^e^	0.24 ± 0.05^b^	6.64 ± 0.16^g^
Sifeng	13.05 ± 0.01^e^	0.35 ± 0.01^e^	83.03 ± 3.37^a^	11.00 ± 0.00^b^	29.14 ± 0.54^e^	77.82 ± 2.99^d^	38.5 ± 0.00^e^	371.15 ± 1.78^e^	99.58 ± 0.04^c^	−0.02 ± 0.04^f^	8.64 ± 0.08^a^
Xuejian WC	13.81 ± 0.01^d^	0.46 ± 0.06^bcd^	79.62 ± 1.90^a^	12.00 ± 0.39^a^	32.68 ± 0.42^ab^	82.40 ± 1.29 cd	51.75 ± 0.75^a^	452.57 ± 6.85^a^	99.44 ± 0.05^d^	0.34 ± 0.05^a^	6.58 ± 0.07^gh^
Sany	12.36 ± 0.11^f^	0.60 ± 0.01^a^	77.14 ± 2.55^a^	12.48 ± 0.16^a^	32.35 ± 0.35^abc^	82.39 ± 1.36 cd	47.25 ± 0.75^b^	418.01 ± 3.29^c^	98.64 ± 0.05^g^	0.26 ± 0.05^b^	7.88 ± 0.13^b^
Golden Arowana	13.91 ± 0.01 cd	0.50 ± 0.06^abc^	79.41 ± 0.72^a^	11.99 ± 0.08^a^	30.24 ± 0.26^de^	88.42 ± 1.09^b^	44.25 ± 0.25^c^	416.84 ± 7.93^c^	99.42 ± 0.04^d^	0.10 ± 0.00 cd	7.62 ± 0.07^d^
Wudeli	13.11 ± 0.04^e^	0.41 ± 0.03^cde^	81.39 ± 0.50^a^	10.91 ± 0.02^b^	27.40 ± 0.07^f^	94.71 ± 0.16^a^	42.00 ± 0.00^d^	424.18 ± 6.00^bc^	100.02 ± 0.04^a^	0.02 ± 0.04^ef^	6.80 ± 0.11^f^
Jinyuan	14.65 ± 0.16^a^	0.58 ± 0.04^a^	76.90 ± 1.19^a^	12.45 ± 0.22^a^	33.03 ± 0.50^ab^	78.19 ± 0.58^d^	47.00 ± 0.50^b^	427.15 ± 14.03^bc^	98.68 ± 0.04^g^	0.26 ± 0.05^b^	7.66 ± 0.10 cd
Fengzheng LG	14.17 ± 0.03^b^	0.53 ± 0.02^ab^	78.72 ± 2.19^a^	12.11 ± 0.07^a^	33.13 ± 0.10^a^	83.41 ± 2.44^bcd^	51.50 ± 0.50^a^	445.49 ± 3.43^ab^	99.92 ± 0.04^b^	0.06 ± 0.05^de^	6.44 ± 0.05^h^
Fengzheng HG	14.14 ± 0.03^bc^	0.38 ± 0.01d^e^	82.35 ± 1.82^a^	8.35 ± 0.05^c^	21.89 ± 0.61^f^	95.26 ± 1.92^a^	38.25 ± 0.25^e^	367.04 ± 7.06^e^	98.96 ± 0.05^f^	0.16 ± 0.05^c^	7.56 ± 0.05^d^

Note

All the results are mean ± *SD* of three individual determinations. Values followed by different letters in a column are significantly different at 0.05 probability level.

Abbreviations: GI, gluten index; SIG, swelling index of glutenin; WG, wet gluten; ZeSV, zeleny sedimentation value.

### 3.2 Rheological properties

Rheological index of wheat dough is an important basis for predicting the quality of final wheat‐based products. Farinographic property reflects the dynamic changes of the consistency of dough during stirring (Mis et al., 2012). The FWA ranged between 59.8% and 65.1% (Table 2). The variation in FWA might be attributed not only to the type of protein but also to the amount of starch (Sarker et al., 2008). The values of dough development time (DDT), dough stability time (DST), and degree of softening (DOS) differed widely from 1.6 to 21.8 min, 3.4 to 23.8 min, and 26 to 75 BU, respectively. The Wudeli flour exhibited the strongest dough strength as evident from the highest DDT, the highest DST, and lower DOS values. Conversely, Fengzheng LG flour had the weakest dough strength for the lowest DDT and DST and higher DOS. This might be attributed to the higher GI and SIG of Wudeli flour and lower GI of Fengzheng LG flour, indicating that the gluten strength and quality of Wudeli flour were better. The result was coincided with that of Gulia and Khatkar (2013) who found that the dough development time and dough stability obtained by Mixolab test showed a significant positive correlation with gluten quality.

**TABLE 2:** Rheological properties of different wheat flours

Varieties	Farinographic property				Extensographic property			
	FWA (%)	DDT (%)	DST (min)	DOS (BU)	A (cm^2^)	R (BU)	E (mm)	R/E
Xiangxue HG	65.1 ± 0.3^a^	2.8 ± 0.3^e^	4.9 ± 0.2^ef^	71±1^a^	57±3^efg^	222±19^fg^	162±7^abc^	1.4 ± 0.1^d^
Xiangmanyuan	61.4 ± 0.1^d^	3.0 ± 0.1^de^	5.0 ± 0.0^ef^	69±3^a^	49±1^g^	201±0^g^	174±4^ab^	1.2 ± 0.1^d^
Xuejian MP	65.0 ± 0.0^a^	5.4 ± 0.1^b^	12.1 ± 0.6^bc^	32±1 cd	91±7^a^	309±4^bc^	171±17^abc^	1.9 ± 0.2^bc^
Sifeng	60.4 ± 0.1^e^	6.2 ± 0.2^b^	7.8 ± 0.8d^e^	75±2^a^	51±0^fg^	252±14^def^	128±9^de^	2.0 ± 0.2^bc^
Xuejian WC	65.0 ± 0.2^a^	2.3 ± 0.0^ef^	15.8 ± 1.7^b^	26±4^d^	90±5^a^	322±5^ab^	166±6^abc^	2.0 ± 0.1^bc^
Sany	62.6 ± 0.1^c^	3.9 ± 0.1 cd	6.2 ± 0.2^def^	73±2^a^	65±0^cde^	262±0^de^	146±0 cd	1.8 ± 0.0^c^
Golden Arowana	62.8 ± 0.1^c^	5.4 ± 0.0^b^	14.8 ± 0.2^b^	32±5 cd	82±1^ab^	328±21^ab^	150±5^bcd^	2.2 ± 0.2^b^
Wudeli	63.4 ± 0.2^b^	21.8 ± 1.0^a^	23.8 ± 2.8^a^	44±3^bc^	68±3 cd	357±9^a^	116±1^e^	3.1 ± 0.1^a^
Jinyuan	59.8 ± 0.0^f^	4.3 ± 0.0^c^	9.6 ± 1.0 cd	39±8^bcd^	75±3^bc^	230±7^efg^	178±11^a^	1.3 ± 0.1^d^
Fengzheng LG	60.0 ± 0.1^f^	1.6 ± 0.1^f^	3.4 ± 1.2^f^	74±8^a^	60±3^def^	341±18^ab^	113±0^e^	3.1 ± 0.2^a^
Fengzheng HG	62.6 ± 0.0^c^	4.2 ± 0.4^c^	8.2 ± 1.0^de^	50±5^b^	73±0^bc^	279±6 cd	158±4^abc^	1.8 ± 0.0^c^

Note

All the results are mean ± *SD* of three individual determinations. Values followed by different letters in a column are significantly different at 0.05 probability level.

Abbreviations: A, area under the curve, total energy; DDT, dough development time; DOS, degree of softening; DST, dough stability time; E, extensibility; FWA, flour water absorption; R, resistance; R/E, ratio of resistance to extensibility.

Extensographic properties reflect the resistance to stretching of dough. As shown in Table 2, the area under the curve (A), the R, the E, and the R/E ratio varied from 49 to 91 cm^2^, 201 to 357 BU, 113 to 178 mm, and 1.2 to 3.1, respectively. Higher R values, lower E values, and highest R/E ratios were found in Wudeli and Fengzheng LG flours, indicating that these two flour brands had higher strength and elasticity while lower extensibility of gluten. Oppositely, the lowest R and R/E and higher E values were observed for Xiangmanyuan flour, suggesting that Xiangmanyuan flour exhibited weaker gluten strength. These differences in dough properties were observed due to the variations in gluten contents of wheat flours (Kaur et al., 2016).

### 3.3 Pasting properties

Pasting properties, analyzed by a rapid visco analyzer (RVA), indicate the swelling and crystal collapse of starch granules during heating and cooling process in a system (Hormdok & Noomhorm, 2007). As shown in Table 3, the values of peak viscosity (PV), trough viscosity (TV), breakdown viscosity (BDV), final viscosity (FV), and setback viscosity (SBV) ranged from 2,440.0 to 3,274.5 cP, 1624.0 to 2,495.0 cP, 530.0 to 969.5 cP, 2,894.0 to 3,803.5 cP, and 1,204.5 to 1,363.0 cP, respectively. Flour from Sany brand exhibited the lowest PV, TV, and FV values among all the flours. High‐protein content of Sany flour (12.48%) might be responsible for this phenomenon. Singh et al. (2014) and Shevkani et al. (2015) demonstrated that the presence of more proteins could stabilize the continuous matrix, protected the integrity of starch granules, and decreased the paste viscosity. The value of BDV indicates the shear resistance of starch at high temperature or the stability of starch paste during heating. Obviously, Fengzheng LG flour showed the lowest BDV while Fengzheng HG flour showed the highest. Wudeli flour exhibited a higher retrogradation tendency as evident from the highest SBV values while Jinyuan flour had the lowest. Differences in starch contents, especially the amylose contents, might explain this phenomenon. Noda et al. (2001) found that higher amylose content was the major factor contributing to higher SBV. The PT provides an indication of the lowest temperature required to gelatinize starch as well as the temperature at which the viscosity increases during heating (Singh et al., 2011). PT was observed to be 68.5–87.6℃. The highest temperature was found for Fengzheng LG flour and the lowest for Xiangmanyuan, Wudeli, and Fengzheng HG flours. The high PT for Fengzheng LG flour showed that its starch was highly resistant to expansion and rupture because of the higher protein content in this flour (Shevkani et al., 2015; Singh et al., 2014).

**TABLE 3:** Pasting properties of different wheat flours

Varieties	PV (cP)	TV (cP)	BDV (cP)	FV (cP)	SBV (cP)	PT (^o^C)
Xiangxue HG	2,739.0 ± 37.0^e^	2098.5 ± 50.5^c^	640.5 ± 13.5^de^	3,371.0 ± 28.0^c^	1,272.5 ± 22.5^abcd^	69.3 ± 0.0^cde^
Xiangmanyuan	2,881.5 ± 44.5^d^	2,280.5 ± 46.5^b^	601.0 ± 2.0^ef^	3,580.5 ± 48.5^b^	1,300.0 ± 2.0^abc^	68.5 ± 0.1^e^
Xuejian MP	2,517.0 ± 27.0^fg^	1902.5 ± 26.5^e^	614.5 ± 0.5^ef^	3,114.0 ± 26.0^f^	1,211.5 ± 0.5 cd	70.5 ± 0.4^b^
Sifeng	3,183.0 ± 1.0^b^	2,495.0 ± 14.0^a^	688.0 ± 13.0 cd	3,791.0 ± 13.0^a^	1,296.0 ± 27.0^abcd^	68.9 ± 0.4^de^
Xuejian WC	2,492.5 ± 22.5^fg^	1992.5 ± 24.5^de^	570.0 ± 2.0^fg^	3,144.0 ± 33.0^ef^	1,221.5 ± 8.5 cd	69.8 ± 0.4^bcd^
Sany	2,440.0 ± 4.0^g^	1624.0 ± 17.0^f^	816.0 ± 21.0^b^	2,894.0 ± 11.0^g^	1,270.0 ± 28.0^bcd^	69.7 ± 0.4^bcd^
Golden Arowana	3,016.0 ± 15.0^c^	2,291.0 ± 4.0^b^	725.0 ± 11.0^c^	3,604.5 ± 28.5^b^	1,313.5 ± 24.5^ab^	70.1 ± 0.0^bc^
Wudeli	3,157.0 ± 6.0^b^	2,440.5 ± 22.5^a^	716.5 ± 28.5^c^	3,803.5 ± 30.5^a^	1,363.0 ± 53.0^a^	68.5 ± 0.1^e^
Jinyuan	2,713.5 ± 27.5^e^	2015.0 ± 41.0 cd	698.5 ± 13.5^c^	3,219.5 ± 18.5^de^	1,204.5 ± 22.5^d^	69.8 ± 0.4^bcd^
Fengzheng LG	2,574.0 ± 38.0^f^	2044.0 ± 56.0^c^	530.0 ± 18.0^g^	3,216.0 ± 20.0^d^	1,217.0 ± 36.0 cd	87.6 ± 0.4^a^
Fengzheng HG	3,274.5 ± 10.5^a^	2,305.0 ± 34.0^b^	969.5 ± 23.5^a^	3,640.5 ± 10.5^b^	1,335.5 ± 23.5^ab^	68.5 ± 0.0^e^

Note

All the results are mean ± *SD* of three individual determinations. Values followed by different letters in a column are significantly different at 0.05 probability level.

Abbreviations: BDV, breakdown viscosity; FV, final viscosity; PT, pasting temperature; PV, peak viscosity; SBV, setback viscosity; TV, trough viscosity.

### 3.4 Quality of hollow noodles made by different wheat flour

The cooking properties include the optimum CT, NWA, and cooking loss (CL). Overcooked noodles are undesirable for too soft and sticky taste. In contrast, a raw flour taste and hard texture are found for undercooked noodles during biting and, hence, cooking time is a key indicator for texture quality of noodles (Park & Baik, 2004). As shown in Table 4, the optimum CT of hollow noodles made by different wheat flours ranged between 3.38 and 4.83 min. Noodles from Wudeli flour required longer time to cook while those from Sifeng and Jinyuan flours took less time. The NWA varied from 155.07% to 179.73%, being the lowest for Golden Arowana flour and the highest for Xiangmanyuan flour. These differences might be attributed to the changes in protein and damaged starch contents of flours (Park & Baik, 2004). A lot of research had reported that starch and protein, as two major components of wheat flour largely determined the processing and product quality of many wheat‐based foods (Fu, 2008). The CL is assessed based on the total solids existed in cooking water. The values of CL ranged from 3.60% to 5.71%. Golden Arowana flour displayed the highest values while Xuejian MP flour showed the lowest CL values.

**TABLE 4:** Cooking, textural properties, and sensory evaluation of hollow noodles made of different wheat flours

Varieties	CT (min)	NWA (%)	CL (%)	HD (g)	AD (g)	SP (ratio)	CO (ratio)	Ten‐*F* (g)	Ten‐D (mm)	Hol‐R (%)	Color	Firmness	Viscoelasticity	Smoothness	Flavor
Xiangxue HG	4.75 ± 0.01^a^	173.59 ± 1.44^ab^	4.20 ± 0.04^d^	5,204.14 ± 123.53^a^	191.64 ± 18.86^a^	0.93 ± 0.00^a^	0.72 ± 0.00^ab^	12.85 ± 2.00^ab^	61.19 ± 13.48^a^	63.83 ± 3.00^a^	8.28 ± 0.36^a^	6.90 ± 0.82^a^	7.30 ± 0.93^a^	5.85 ± 0.00^a^	7.65 ± 0.45^ab^
Xiangmanyuan	3.48 ± 0.02^b^	179.73 ± 4.98^a^	3.66 ± 0.04^gh^	4,665.88 ± 220.64^ab^	152.40 ± 58.05^ab^	0.92 ± 0.00^ab^	0.73 ± 0.01^a^	13.14 ± 1.66^ab^	60.92 ± 12.22^a^	23.00 ± 3.00^cde^	7.74 ± 0.92^bc^	6.45 ± 0.68^a^	7.30 ± 0.51^a^	5.25 ± 0.56^a^	7.43 ± 0.39^ab^
Xuejian MP	3.46 ± 0.13^b^	160.18 ± 1.72^cde^	3.60 ± 0.01^h^	4,880.12 ± 529.77^ab^	119.61 ± 51.65^ab^	0.89 ± 0.02^abc^	0.68 ± 0.03^b^	12.86 ± 0.11^ab^	57.46 ± 13.73^a^	42.00 ± 6.00^b^	5.76 ± 0.72^e^	6.90 ± 0.50^a^	7.60 ± 0.14^a^	5.70 ± 0.21^a^	7.88 ± 0.39^a^
Sifeng	3.38 ± 0.04^b^	163.48 ± 0.20^bcde^	3.88 ± 0.01^ef^	4,776.34 ± 202.11^ab^	111.00 ± 6.45^ab^	0.92 ± 0.02^ab^	0.70 ± 0.02^ab^	12.91 ± 1.43^ab^	59.58 ± 12.08^a^	8.50 ± 3.50^e^	8.28 ± 0.67^a^	6.75 ± 0.98^a^	7.00 ± 0.71^a^	5.40 ± 0.64^a^	6.98 ± 0.39^b^
Xuejian WC	3.50 ± 0.08^b^	156.55 ± 2.64^de^	3.94 ± 0.04^e^	4,697.57 ± 130.57^ab^	91.67 ± 1.67^b^	0.86 ± 0.01 cd	0.70 ± 0.01^ab^	14.33 ± 0.37^ab^	59.70 ± 5.63^a^	38.00 ± 3.00^bc^	6.30 ± 0.00^cde^	7.20 ± 0.57^a^	7.70 ± 0.14^a^	5.10 ± 0.76^a^	7.43 ± 0.39^ab^
Sany	3.58 ± 0.08^b^	166.48 ± 1.62^bcde^	3.76 ± 0.08^fg^	5,058.21 ± 0.00^ab^	92.98 ± 9.52^b^	0.84 ± 0.00^d^	0.70 ± 0.01^ab^	14.77 ± 1.15^a^	62.71 ± 20.03^a^	43.00 ± 2.00^b^	7.02 ± 0.36^bcd^	6.75 ± 0.98^a^	8.00 ± 0.37^a^	5.55 ± 0.21^a^	7.65 ± 0.45^ab^
Golden Arowana	3.72 ± 0.05^b^	155.07 ± 0.17^e^	5.71 ± 0.03^a^	4,825.45 ± 360.04^ab^	110.55 ± 1.80^ab^	0.89 ± 0.00^bc^	0.70 ± 0.00^ab^	12.77 ± 0.92^ab^	56.94 ± 6.78^a^	24.00 ± 3.00^cde^	6.66 ± 0.92^bcde^	7.05 ± 0.38^a^	7.70 ± 0.37^a^	5.25 ± 0.56^a^	7.43 ± 0.39^ab^
Wudeli	4.83 ± 0.17^a^	161.51 ± 4.21^bcde^	5.01 ± 0.07^b^	4,806.63 ± 21.04^ab^	130.94 ± 26.45^ab^	0.91 ± 0.02^ab^	0.73 ± 0.00^a^	13.46 ± 0.08^ab^	82.78 ± 10.69^a^	20.50 ± 3.50^de^	7.38 ± 0.88^abc^	7.50 ± 0.50^a^	7.90 ± 0.37^a^	6.00 ± 0.56^a^	7.20 ± 0.00^ab^
Jinyuan	3.38 ± 0.29^b^	169.91 ± 2.05^abc^	5.02 ± 0.04^b^	4,231.15 ± 288.63^b^	94.16 ± 6.54^b^	0.91 ± 0.01^abc^	0.72 ± 0.01^ab^	10.66 ± 1.16^b^	56.30 ± 2.53^a^	37.50 ± 7.50^bc^	5.94 ± 1.22^de^	6.60 ± 0.50^a^	7.30 ± 0.62^a^	5.70 ± 0.21^a^	7.20 ± 0.64^ab^
Fengzheng LG	3.79 ± 0.13^b^	163.64 ± 8.94^bcde^	4.53 ± 0.03^c^	4,661.17 ± 355.57^ab^	153.23 ± 0.89^ab^	0.92 ± 0.01^ab^	0.71 ± 0.00^ab^	12.23 ± 0.52^ab^	67.34 ± 2.75^a^	14.00 ± 8.00^e^	7.74 ± 0.72^ab^	6.60 ± 0.50^a^	7.60 ± 0.37^a^	5.25 ± 0.56^a^	7.43 ± 0.39^ab^
Fengzheng HG	4.54 ± 0.04^a^	168.31 ± 4.11^abcd^	3.82 ± 0.05^ef^	4,743.87 ± 220.92^ab^	102.69 ± 17.07^ab^	0.88 ± 0.02^bcd^	0.71 ± 0.01^ab^	12.94 ± 0.53^ab^	72.04 ± 13.00^a^	32.50 ± 6.50^bcd^	6.66 ± 0.92^bcde^	7.05 ± 0.38^a^	7.30 ± 0.99^a^	5.25 ± 0.56^a^	7.65 ± 0.45^ab^

Note

All the results are mean ± *SD* of three individual determinations. Values followed by different letters in a column are significantly different at 0.05 probability level.

Abbreviations: AD, adhesiveness; CL, cooking loss; CO, cohesiveness; CT, cooking time; HD, hardness; Hol‐R, hollow rate; NWA, noodle water absorption; SP, springiness; Ten‐D, tensile distance; Ten‐F, tensile force.

The instrumental texture test of cooked noodles, as a valuable research tool, is suitable for the quality monitoring of noodles (Ross, 2006). As shown in table 4, textural properties were influenced significantly by flour brands (*p* < .05). The hardness (HD), adhesiveness (AD), springiness (SP), and cohesiveness (CO) varied from 4,231.15 to 5,204.14 g, 91.67 to 191.64 g, 0.84 to 0.93, and 0.68 to 0.73, respectively. Hollow noodles made from Xiangxue HG flour exhibited excellent textural properties as evident from the highest HD, AD, and SP values and higher CO value, while Sany flour had lower AD, the lowest SP, and lower CO values. A high degree of gluten network formation would completely encircled starch granules and formed a compact internal structure of noodle strands, which largely contribute to these results (Park & Baik, 2004).

Moreover, tensile test assesses the ability of noodles to resist breaking, including tensile force (Ten‐F) and tensile distance (Ten‐D). The results of Ten‐F and Ten‐D ranged from 10.66 to 14.77 g and 56.30 to 82.78 mm, respectively (Table 4). Hollow noodles from Sany flour required the maximum force to break and that from Wudeli flour showed the far most tensile distance, while Jinyuan flour exhibited the minimum Ten‐F and Ten‐D.

The Hol‐R of hollow noodles, determined by the proportion of noodles with hollows in total noodles, represents the degree of fermentation. As shown in table 4, the values of Hol‐R ranged from 8.50% to 63.83%, of which hollow noodles made from Xiangxue HG flour had the best fermentation state and hollow noodles from Sifeng flour had the worst. The Hol‐R of the noodles might be related to the composition of the flour (e.g., the sugar consumed by yeast) and the ability of the gluten to hold the gas.

The results of sensory characteristics were reported in the form of scores in table 4. Significant sensory differences existed in hollow noodles made from different flours (*p* < .05). The color scores ranged from 5.76 to 8.28, of which Xiangxue HG and Sifeng flours exhibited the highest and Xuejian MP flour showed the lowest. Although there was no significant differences among these samples (*p* > .05), the scores of firmness, viscoelasticity, and smoothness varied from 6.45 to 7.50, 7.00 to 8.00, and 5.10 to 6.00, respectively. The pleasant degree and uniqueness of the fermented flavor of hollow noodles were determined by the flavor scores. According to table 4, the hollow noodles from Xuejian MP and Xiangxue HG flours had higher flavor scores, which might be attributed to higher Hol‐R of these noodles.

### 3.5 Relationship between wheat flour characteristics and hollow noodle quality

The correlation coefficients between physicochemical properties of different wheat flour and the quality of hollow noodles are presented in Table 5. Among the physicochemical characteristics, the moisture content of flour was negatively correlated to the Ten‐F of cooked hollow noodles (*r* = −.632, *p* < .05). The ash content of flour showed a negative correlation to the color score of cooked hollow noodles (*r* = −.617, *p* < .05), indicating that the flour with higher ash content could darken the brightness and color of noodles. A similar relationship between ash content and noodle color had been reported (He et al., 2004). Although *p* < .05, starch content was positively correlated to color score (*r* = .632). Brighter or whiter appearance might be attributed to more light reflected from more starch granules. A slight negative correlation was observed between protein content and CT (*r* = −.556, *p* > .05), which were not in agreement with the results reported by Kaur et al. (2015, 2016). It has been reported that more proteins in flour might inhibit swelling of starch granules, thus prolonging the cooking time and reducing the cooking loss of noodles (Grzybowski & Donnelly, 1979). In the case of fermented products such as steamed bread, the protein content and quality were positively correlated to the height and volume of steamed bread (De Villiers & Laubscher, 1995; Kim et al., 2019; Ma & Baik, 2016). That was, with the increase of protein content, the total area of gas cells of steamed bread increased. Similarly, noodles with high protein content formed more microholes during fermentation and drying, which might give easy entry for water molecules into the noodles, thus shortening the cooking time. The GI positively related to the NWA (*r* = .661, *p* < .05) while negatively related to the AD of cooked hollow noodles (*r* = −.689, *p* < .05). In addition, the SIG was positively correlated to the viscoelasticity of hollow noodles (*r* = .659, *p* < .05). This might be due to glutenin, which was linked by interchain disulfide bonds, determined dough strength and elasticity (Wieser, 2007). The value of a^*^ showed a negative relationship with the SP (*r* = −.659, *p* < .05) and the color of cooked hollow noodles (*r* = −.738, *p* < .01).

**TABLE 5:** Correction between flour characteristics and hollow noodles properties

Flour characteristics	Cooking properties			Textural properties						Hol‐R	Sensory evaluation				
	CT	CL	NWA	HD	AD	SP	CO	Ten‐F	Ten‐D		Color	Firmness	Viscoelasticity	Smoothness	Flavor
Moisture	−0.061	0.202	0.427	−0.598	−0.596	0.433	0.418	−0.632*	0.195	−0.052	−0.080	−0.378	−0.475	−0.339	−0.090
Ash	−0.494	0.126	−0.205	−0.222	0.321	−0.435	−0.405	−0.116	0.372	0.233	−0.617*	−0.291	0.532	0.076	0.372
Starch	0.375	−0.298	0.371	−0.208	−0.180	0.134	0.390	0.148	−0.231	0.008	0.632*	−0.024	−0.497	−0.160	−0.267
Protein	−0.556	0.238	−0.252	−0.089	0.048	−0.094	−0.230	−0.046	0.526	0.147	−0.197	−0.268	0.390	0.140	−0.026
WG	−0.556	0.066	−0.031	−0.094	−0.139	0.041	−0.100	−0.079	0.588	0.213	−0.053	−0.436	0.202	0.039	0.004
GI	0.047	0.257	−0.689*	−0.208	0.661*	−0.510	−0.439	0.097	−0.435	−0.412	−0.521	0.532	0.452	−0.100	−0.033
ZeSV	−0.472	0.004	−0.404	−0.190	0.189	−0.353	−0.445	0.046	0.305	0.143	−0.517	−0.104	0.520	−0.154	0.305
SIG	−0.329	0.267	−0.522	−0.277	0.233	−0.297	−0.283	0.410	0.074	0.109	−0.514	0.139	0.659*	−0.033	0.086
L*	0.376	0.217	−0.257	0.250	−0.576	0.584	0.201	0.052	−0.423	−0.389	0.518	0.359	0.034	0.098	−0.270
a*	−0.407	−0.294	−0.032	−0.211	0.481	−0.659*	−0.342	0.186	0.426	0.549	−0.738**	−0.082	0.291	−0.196	0.474
b*	−0.235	−0.092	0.357	−0.011	0.193	0.030	0.142	−0.054	0.291	−0.170	0.335	−0.378	−0.514	−0.076	−0.396
FWA	0.370	−0.209	−0.273	0.638*	−0.133	−0.242	−0.303	0.540	−0.037	0.647*	−0.223	0.614*	0.371	0.227	0.615*
DDT	0.496	0.362	−0.235	0.046	0.025	0.171	0.272	0.107	−0.724*	−0.276	0.062	0.672*	0.346	0.610*	−0.354
DST	0.254	0.449	−0.617*	−0.071	0.337	−0.154	−0.084	0.190	−0.410	−0.138	−0.373	0.855**	0.479	0.301	−0.228
DOS	0.087	−0.347	0.596	0.323	−0.499	0.332	0.416	0.081	−0.106	−0.136	0.829**	−0.555	−0.294	0.027	−0.124
A	−0.155	0.173	−0.687*	−0.146	0.550	−0.555	−0.672*	0.056	0.181	0.272	−0.891**	0.482	0.432	−0.077	0.385
R	0.193	0.393	−0.835**	0.064	0.209	−0.245	−0.362	0.204	−0.450	−0.334	−0.266	0.658*	0.629*	−0.049	0.016
E	−0.301	−0.266	0.347	−0.201	0.133	−0.157	−0.118	−0.153	0.609*	0.606*	−0.518	−0.267	−0.250	−0.082	0.415
R/E	0.280	0.389	−0.613*	0.072	−0.001	0.013	−0.077	0.126	−0.622*	−0.546	0.127	0.479	0.488	0.042	−0.231
PV	0.438	0.247	0.069	−0.064	−0.013	0.340	0.326	−0.173	−0.452	−0.469	0.315	0.307	−0.418	0.019	−0.455
TV	0.341	0.289	0.049	−0.120	−0.218	0.595	0.384	−0.259	−0.373	−0.584	0.440	0.237	−0.490	−0.004	−0.587
BDV	0.354	−0.007	0.065	0.096	0.430	−0.426	−0.012	0.122	−0.322	0.086	−0.160	0,250	0.012	0.056	0.126
FV	0.404	0.283	0.057	−0.046	−0.206	0.536	0.407	−0.172	−0.436	−0.559	0.454	0.290	−0.420	0.017	−0.548
SBV	0.601	0.176	0.080	0.323	−0.095	0.111	0.412	0.292	−0.627*	−0.290	0.400	0.465	0.030	0.116	−0.215
PT	−0.013	0.195	0.021	−0.095	−0.472	0.820**	0.133	−0.460	0.110	−0.461	0.478	−0.127	−0.637*	−0.011	−0.480

Note

*p < .05; **p < .01

Abbreviations: A, area under the curve, total energy; AD, adhesiveness; BDV, breakdown viscosity; CL, cooking loss; CO, cohesiveness; CT, cooking Time; DDT, dough development time; DOS, degree of softening; DST, dough stability time; E, extensibility; FV, final viscosity; FWA, flour water absorption; GI, gluten index; HD, hardness; Hol‐R, hollow rate; NWA, noodle water absorption; PT, pasting temperature; PV, peak viscosity; R, resistance; R/E, ratio of resistance to extensibility; SBV, setback viscosity; SIG, swelling index of glutenin; SP, springiness; Ten‐D, tensile distance; Ten‐F, tensile force; TV, trough viscosity; WG, wet gluten; ZeSV, zeleny sedimentation value.

The correlation coefficients between farinographic properties of different wheat flours and the quality of hollow noodles were also presented in Table 5. The FWA was positively correlated to the HD, Hol‐R, firmness, and flavor of hollow noodles (*r* = .638, .647, .614, and .615, respectively, *p* < .05). The DDT exhibited a positive correlation with the firmness and smoothness (*r* = .672 and .610, respectively, *p* < .05), but showed a negative relationship with Ten‐D (*r* = −.724, *p* < .05). Furthermore, the DST was highly positively correlated with the firmness of cooked hollow noodles (*r* = .855, *p* < .01) while was negatively related to the NWA of hollow noodles (*r* = −.617, *p* < .05). Similar results were obtained by previous researches (Ghanate & Annapure, 2019; Yue et al., 2017). A longer stability time could strengthen the gluten of the dough and formed a tight gluten network structure, thus preventing noodles from absorbing water. In addition, the DOS was strongly positively related to the color of hollow noodles (*r* = .829, *p* < .01). The A value was negatively correlated to the NFA and CO of hollow noodles *(r* = −.687 and −.672, respectively, *p* < .05). The R related negatively to the NWA (*r* = −.835, *p* < .01), and the E was positively correlated to the Ten‐D and Hol‐R (*r* = .609 and .606, respectively, *p* < .05). It is demonstrated that the dough with excellent extensibility could provide hollow noodles with better extensibility and fermentation status. The R/E showed negative correlation with the NWA (*r* = −.613, *p* < .05) and Ten‐D (*r* = −.622, *p* < .05). Similar results were obtained by Yue et al. (2017) who found the NWA was negatively correlated to the A, R, and R/E values (*r* = −.671, −.596, and −.583, respectively, *p* < .05).

As shown in Table 5, according to correlation analysis, the SBV of flour was negatively correlated to the Ten‐D of cooked hollow noodles (*r* = −.627, *p* < .05). The PT was positively correlated to the SP (*r* = .820, *p* < .01), while negatively related to the viscoelasticity (*r* = −.637, *p* < .05). Although the correlation coefficient was not significant, the PV related positively to the CT (*r* = .438, *p* ˃ .05) but negatively to the Hol‐R and fermentation flavor (*r* = −.469 and −.455, respectively, *p* ˃ .05). The TV and FV showed similar relationships with CT, Hol‐R, and flavor. Therefore, higher peak, through and final viscosity were not beneficial for the fermentation of hollow noodles.

## 4 CONCLUSIONS

In this study, based on a set of commercial samples, the quality of hollow noodles was associated with physicochemical characteristics, rheological properties including farinograph and extensograph and pasting properties of wheat flours. The CT of hollow noodles was negatively related to protein content while positively related to pasting properties of flour. Extensographic properties had a considerable effect on NWA. The FWA and E were found to be highly correlated to the Hol‐R. A significant positive correlation was observed between SIG and viscoelasticity, PT, and SP. Furthermore, farinographic properties substantially affected the firmness of sensory evaluation. Thus, flour with higher SIG, FWA, E, and PT resulted in hollow noodles with better fermented flavor and better textural quality. So far, there are few studies on the hollow noodles, including formula, quality changes during storage, quality formation mechanism, and further investigations need to be conducted.

## AUTHOR CONTRIBUTIONS

**Xiaoqing Xiong:** Software (equal); Validation (equal); Visualization (equal); Writing‐original draft (lead); Writing‐review & editing (lead). **Chong Liu:** Conceptualization (equal); Resources (lead); Supervision (lead); Validation (equal); Visualization (equal); Writing‐review & editing (supporting). **Mengkun Song:** Writing‐review & editing (supporting). **Xueling Zheng:** Formal analysis (equal); Funding acquisition (lead); Methodology (equal).

## ETHICAL APPROVAL

This study does not involve any human or animal testing. The authors have declared no conflicts of interest for this article.

## Data Availability

The data that support the findings of this study are openly available.
